# Phase Formation of Mg-Zn-Gd Alloys on the Mg-rich Corner

**DOI:** 10.3390/ma11081351

**Published:** 2018-08-03

**Authors:** Lan Luo, Yong Liu, Meng Duan

**Affiliations:** 1 School of Materials Science and Engineering, Nanchang University, Nanchang 330001, China; luolan1190@163.com; 2 Key Laboratory of Lightweight and High Strength Structural Materials of Jiangxi Province, Nanchang University, Nanchang 330031, China; duanmengjiayou@163.com; 3 Key Laboratory of Near Net Forming of Jiangxi Province, Nanchang University, Nanchang 330001, China

**Keywords:** magnesium alloy, Mg-Zn-Gd alloy, quasicrystalline phase (I-phase), W-phase, microstructure

## Abstract

The phase constitutions of as-cast magnesium (Mg)-Zn-Gd alloys (Zn/Gd = 0.25~60, Zn 0~10 at.%, Gd 0~2 at.%, 48 samples) were investigated using X-ray diffraction (XRD), scanning electron microscopy (SEM), and transmission electron microscopy (TEM). The Mg-Zn-Gd phase diagram focused on the Mg-rich corner (with up to 20 at.% Zn, 10 at.% Gd) has been set up. Five regions can be classified as follows: (I) α-Mg+W-phase+(binary compounds), (II) α-Mg+W-phase+I-phase(+binary compounds), (III) α-Mg+I-phase(+binary compounds), (IV) α-Mg+binary compounds, and (V) α-Mg. The phase diagram has been verified by solidification behaviors observation using differential thermal analysis (DTA). Moreover, the structures of I-phase and W-phase in the alloy were explored in details. In terms of the Hume-Rothery rules, I-phase is confirmed as FK-type quasicrystalline with a chemical stoichiometry as Mg_30±1_Zn_62_Gd_8±1_ (at.%). The composition and lattice parameter a W-phase (fcc structure, $m\bar{3}m$) are affected by the composition of Mg-Zn-Gd alloys, especially by the Zn/Gd ratio of alloys. This work would be instructive for the design of Mg-Zn-Gd alloys to obtain the phase components, and then selected the strengthening ways, which could adjust its mechanical properties.

## 1. Introduction

Magnesium (Mg) alloys are very attractive for automobile applications because of their light weight, energy saving, and beneficial properties for environment [1]. Among them, the Mg-Zn-Gd system seems particularly promising in that it offers a unique opportunity to produce both prismatic and basal precipitate planes [2], the combination of which can lead to a maximum impediment to propagating dislocations and twins, and hence exceptional mechanical properties. But, there is very limited information on the phase formation in this system. Such information is critically needed not only for designing alloys with further improvement in strength, but also for any insightful understanding of the texture weakening effect of Gd in dilute Mg-Zn-Gd alloys and the effects of intermetallic phases, such as quasicrystalline on mechanical properties in more concentrated Mg-Zn-Gd extrusion alloy [3].

Icosahedral quasicrystalline phase (I-phase) and long-period stacking ordered (LPSO) structures have been widely explored for the strengthening effect. Although there is inconsistency with regard to the stability of I-phase, it has still been admitted as a very important strengthening way for Mg-Zn-RE system, such as Mg-Zn-Y [4,5,6,7,8,9,10], Mg-Zn-Gd [11,12,13,14,15], Mg-Zn-Er [16,17], and Mg-Zn-Ho [18,19]. During the hot deformation process (i.e., hot rolling, extrusion, equal channel angular extrusion (ECAE), and severe hot rolling), I-phase particles are claimed to be stable against coarsening, and can retain strength at high temperatures due to strong interface bonding strength [5,6,7,8]. In addition, the alloys containing LPSO structures, a thickness of a single unit cell height and having an extraordinarily large aspect ratio structure, exhibit superior mechanical properties. After rapidly solidified powder metallurgy (RS P/M), Mg-1Zn-2Y (at.%) [20,21], and Mg-Zn-(Y, Dy, Ho, Er) [22,23,24,25] alloys would obtain LPSO structures. Mg-Zn-Gd alloy could both form this staking from the supersaturated solid solution phase within elevated temperature, as reported by M. Yamasaki et al. [26,27]. W-phase (${\left(100\right)}_{W^{\prime }}$, Mg_3_ RE_2_Zn_3_) in Mg-Zn-RE alloy system was first reported by Padezhnova et al. [28], which could be easily formed in the solidification of alloy. W-phase basically has no strengthening effect on Mg alloy [29] because of its limited structural symmetry and weak adhesive strength with the Mg matrix [30]. A research of Xu [31] reveals that a small volume fraction of W phase (below 13.8%) is beneficial to the ductility of Mg-Zn-Y-Zr alloy. Thus, the precipitation of the W-phase in Mg-Zn-RE alloys is still worthy to be investigated.

In this paper, X-ray diffraction (XRD), scanning electron microscopy (SEM), and transmission electron microscopy (TEM) were employed to study the typical microstructures and the phase constitutions of Mg-Zn-Gd alloys in as-cast. The phase formation regions could be explored and validated by samples with up to 10 at.% Gd and 20 at.% Zn. The DTA experimental observation was used to verify the phase constitution results. Moreover, the intermetallic phase structures in the as-cast alloy, such as I-phase and W-phase, were discussed in detail.

## 2. Material and Methods

**Figure 1:**
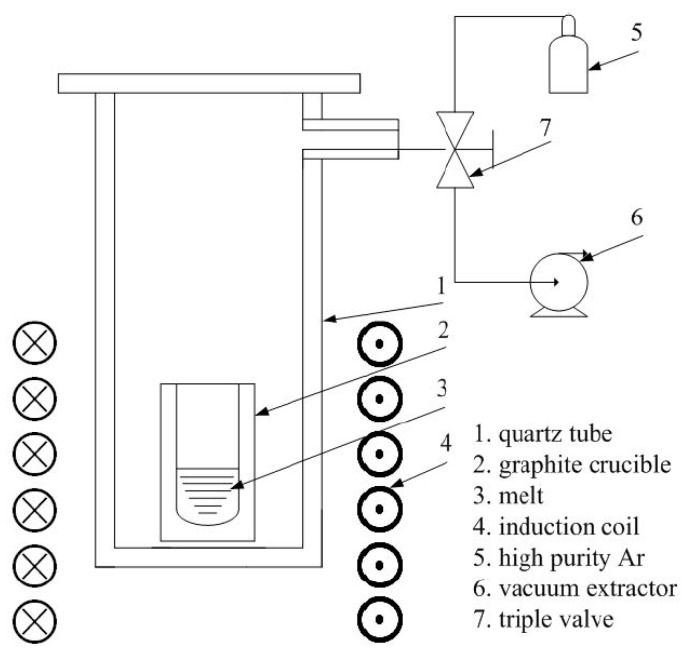
Sketch map of the melting device.

In total, 48 ternary Mg-Zn-Gd samples were prepared (with up to 2 at.% Gd, 10 at.% Zn, 0.25~60 Zn/Gd atomic ratio). The sample compositions are shown in Table 1, together with analytical methods. All of the samples were produced from high purity Mg (99.9 wt %), Zn (99.99 wt %), and Gd (99.99 wt %) by high-frequency induction melting in the graphite crucible and then casting into a steel mold under an argon atmosphere. Typical weight of these samples was 50 g. In order to prevent the vaporization of Mg and Zn, the graphite crucibles containing the weighted raw materials were sealed into quartz tubes under a rarified argon atmosphere. The sketch map of the melting device was shown in Figure 1.

The phase constitutions for all of the alloys were examined by X-ray diffraction (XRD, D8 Advance-Bruker, Karlsruhe, Germany) at 40 kV and 40 mA with the Cu-Kα radiation. For the typical alloys, the microstructures were characterized by scanning electron microscopy (SEM, JEM-6460, JEOL, Tokyo, Japan or FEI Sirion-200, Hillsboro, OR, USA), equipped with an energy-dispersal X-ray spectroscopy (EDS, Oxford X-maxn, Oxford, UK). In special alloys, some phases were further characterized by transmission electron microscopy (TEM, JEM-2010 equipped with Oxford inca EDS system) (JEOL, Tokyo, Japan) to determine the crystal structure. For TEM examinations, the discs of 3 mm diameter and 0.1 mm thickness were prepared from the bulk sample by electric spark machining cutting, and then mechanical thinning, twin-jet elector-polishing to 0.1 mm, followed by ion milling using a Gatan precision ion polishing system (PIPS). The typical alloys were also investigated by differential thermal analysis (DTA) (MDSC2910, Perkin Elmer, Waltham, MA, USA) at a heating/cooling rate of 0.17 K/s. Helium at a 2 L/h flow rate was applied as the analysis chamber gas. The overall uncertainty of DTA measurements for temperature determination was less than 3 K.

## 3. Results

### 3.1. Phase Formation of Mg-Zn-Gd Alloy on the Mg-rich Corner

The phase identification in this work was based mainly by XRD, but SEM/EDS/TEM were also conducted on selected alloys to confirm the phase identification. The phases identified in all 48 alloys are given in Table 1 (first sort by the Zn descending, then sort by Gd descending). Some experimental findings that were reported in the literature [32,33,34] were also given for comparison.

**Figure 2:**
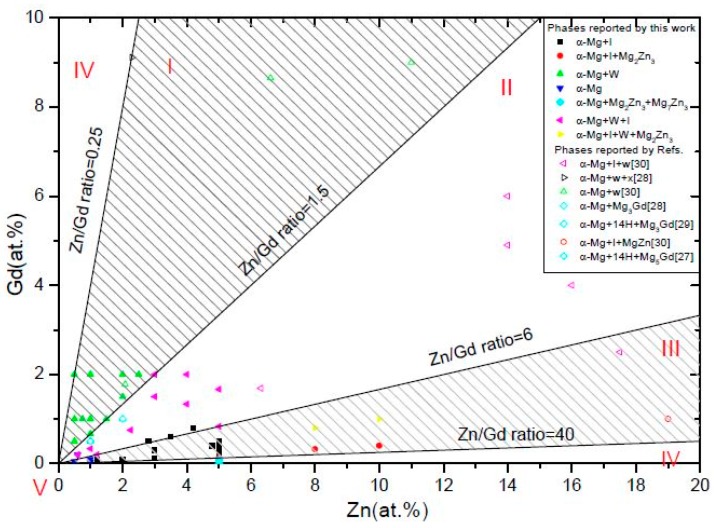
Phase formation of Mg-Zn-Gd alloys on the Mg-rich corner (Zn 0~20 at.%, Gd 0~10 at.%): (I) α-Mg+W-phase(+binary compounds), (II) α-Mg+W-phase+I-phase(+binary compounds), (III) α-Mg+I-phase(+binary compounds), (IV) α-Mg+binary compounds, and (V) α-Mg.

Figure 2 has summarized the phase formation information for Mg-Zn-Gd system focusing on the Mg-rich corner with up to 10 at.% Gd and 20 at.% Zn. During the casting process, the melt was poured into a permanent mold with a comparatively faster cooling rate, which resulted in unequilibrium phase formation. The intermetallic phases that were observed in as-cast samples were mainly I-phase, W-phase, and binary Mg-Zn, Mg-Gd compound, but without any Zn-Gd compound or LSPO structures. So, the phase formation would be a little different from the equilibrium Mg-Gd-Zn ternary phase diagram, as presented by J. Grobner [34] or H.Y. Qi [35]. Five regions can be classified as follows:

(i) α-Mg+W-phase(+binary compounds),
(ii) α-Mg+W-phase+I-phase(+binary compounds),
(iii) α-Mg+I-phase(+binary compounds),
(iv) α-Mg+binary compounds, and
(v) α-Mg.

The region division is mainly according to the X-ray diffraction results for not all of the alloys have been further characterized by TEM/SEM/EDS. Some binary compounds in certain alloys may not be detected for XRD analysis sensitivity.

#### 3.1.1. The Typical Microstructures and Phase Structures

**Figure 3:**
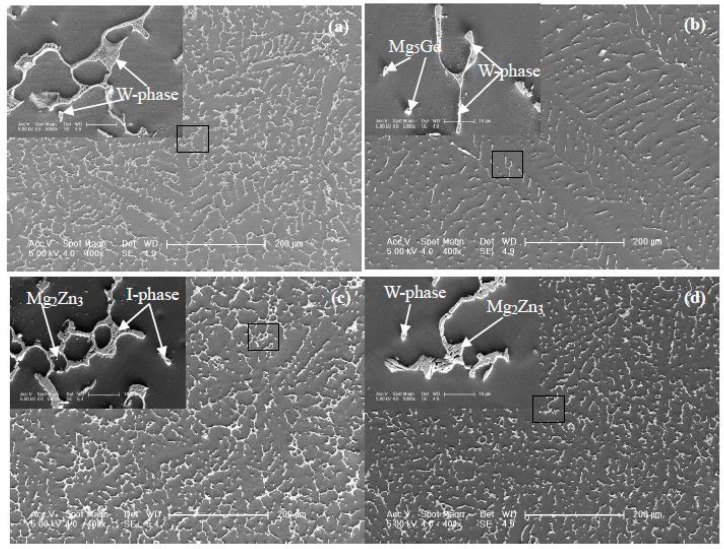
Second electronic image (SEI) of Mg-Zn-Gd alloys (Zn/Gd ratio, sample No.): (**a**) Mg-1Zn-2Gd (0.5, Sample 35), (**b**) Mg-1Zn-0.333Gd (3, Sample 39), (**c**) Mg-5Zn-0.278Gd (18, Sample 10), and (**d**) Mg-5Zn-0.083Gd (60, Sample 14).

With the typical alloys (Samples as 35, 39, 10, and 14), the microstructures were investigated by SEM. From the second electronic image (SEI, Figure 3), Gd content effect on the microstructure of Mg-Zn-Gd alloys could be seen. With a higher Gd content, a larger amount of dendritic phase and more closed network microstructure would exist in alloys. While, with a lower Gd content, a dispersed and granular microstructure would exhibit. The microstructures of Mg-Zn-Gd alloys are coherent with our previous work [11,12,13,14,15] and literature [32,33]. To further identify the phase stoichiometry, EDS was carried out and listed in Table 2. For the Mg-1Zn-2Gd alloy (Sample 35, Figure 3a), the particle and dendritic phases have the similar Zn/Gd ratio and verified as W-phase (Mg_90.85±1.0_Zn_3.95±1.0_Gd_5.2±1.0_, at.%, confirmed by XRD analysis later). The matrix behaves as a supersaturated solid solution phase of α/-Mg. One lamellar phase, with 14H-phase, would appear from this matrix in by heat treatment, and it exhibits thermodynamic equilibrium and thermal stability [26,27]. The same stacking sequence was observed in annealed rapidly solidified (RS) Mg-1Zn-2Y alloys [23]. For Mg-1Zn-0.333Gd alloy (Sample 39, Figure 3b), there exists some granular phase verified as Mg_5_Gd and dendritic phase verified as W-phase (Zn/Gd ≈ 2, confirmed by TEM analysis later). For Mg-5Zn-0.278Gd alloy (Sample 10, Figure 3c), it reveals a larger amount of dendritic phase ascertained as I-phase with a composition of Mg_56.1±1.0_Zn_40±1.0_Gd_3.9±1.0_ (at.%, Zn/Gd = 10.2, confirmed by TEM analysis later). Mg_2_Zn_3_ with finely size of about 0.6 μm could also be founded in this alloy. For the Mg-5Zn-0.083Gd alloy (Sample 14, Figure 3d), it mainly contained Mg_2_Zn_3_ besides a small amount of W-phase (confirmed by XRD analysis later).

**Figure 4:**
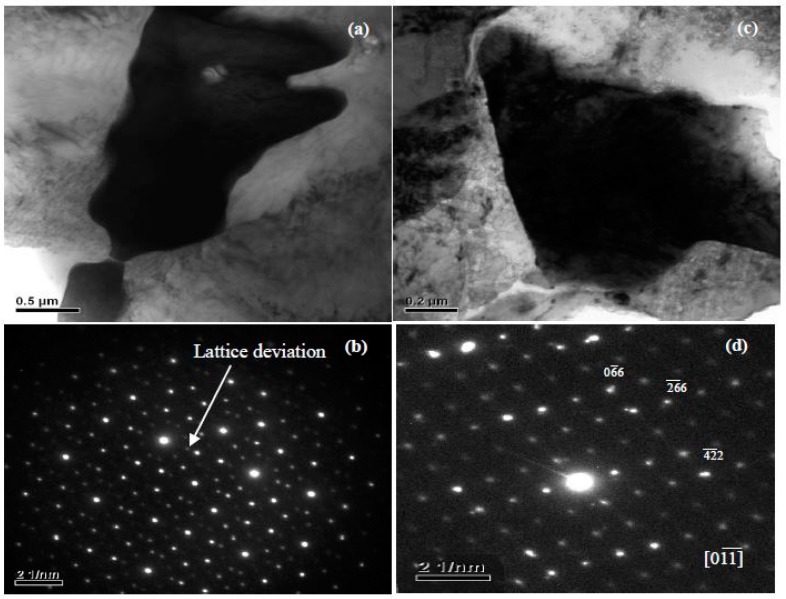
Identification of I-phase with dendritical shape by transmission electron microscopy (TEM) in Mg-5Zn-0.278Gd alloy (Sample 10): (**a**) bright field (BF) image, (**b**) five-fold axes selected area electron diffraction (SAED) pattern, there exists some lattice deviations as pointed with white arrow which means the liner phason strain in I-phase. The I-phase has a composition of approximate Mg_30±1_Zn_62_Gd_8±1_ (at.%) and is identified to have point group symmetry $m\bar{3}\bar{5}$, which is inconsistent with lattice transitions. Identification of W/-phase with triangular shape by TEM in Mg-5Zn-0.278Gd alloy (Sample 10): (**c**) BF image, (**d**) SAED pattern along $\left[0\bar{1}\bar{1}\right]$ zone axis. The W′-phase has a composition of approximate Mg_94±1_Zn_5.47±0.5_Gd_0.53±0.1_ (at.%) and is identified to have a face centered cubic (fcc) structure with a lattice parameter of *a* = 2.3529 nm.

**Figure 5:**
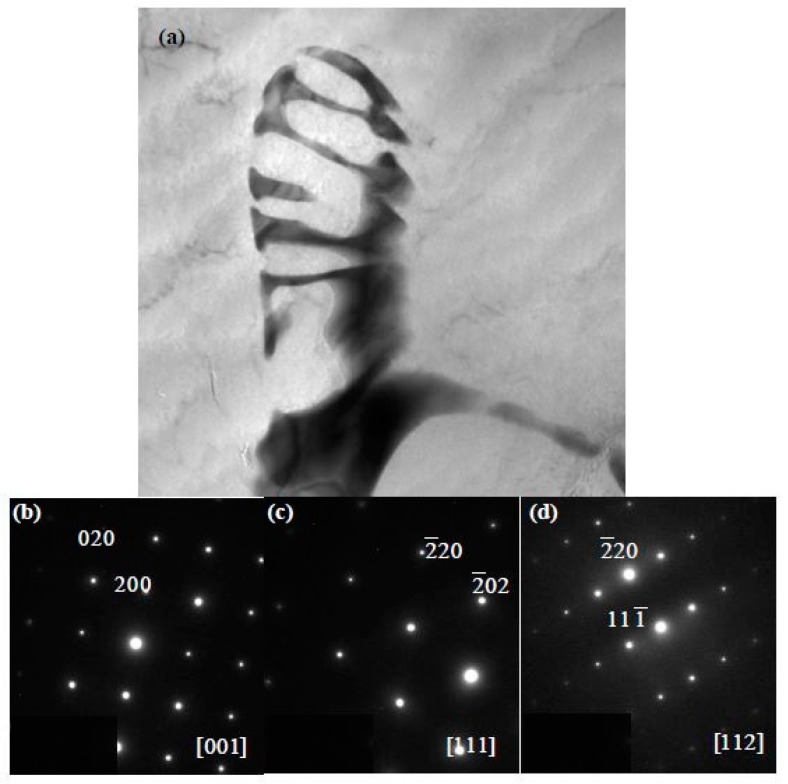
Identification of W-phase with the skeleton morphology by TEM in Mg-1Zn-0.333Gd alloy (Sample 39): (**a**) BF image, and (**b**–**d**) SAED patterns along [001], [111] and [112] zone axis, respectively. The W-phase has a composition of approximate Mg_31_Zn_45_Gd_24_ (Zn/Gd = 1.9, at.%) and it is identified to have a face centered cubic (fcc) structure with a lattice parameter of *a* = 0.6927 nm.

**Figure 6:**
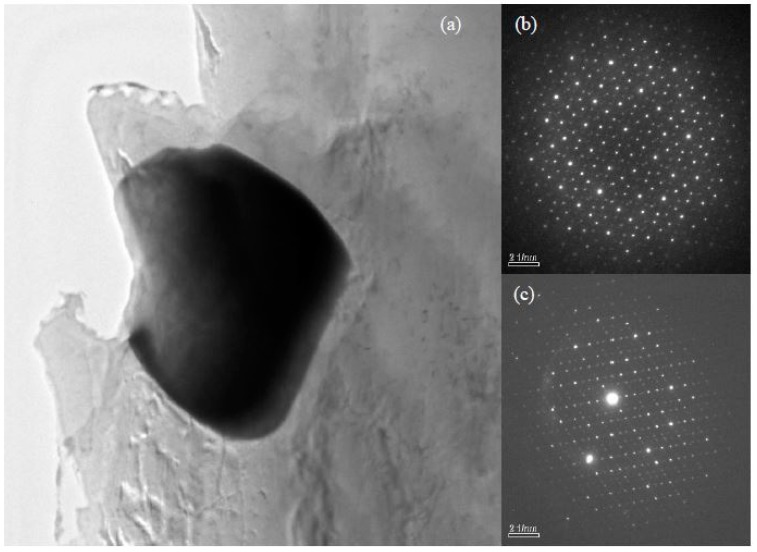
Identification of I-phase with particle shape by TEM in Mg-1Zn-0.333Gd alloy (Sample 39): (**a**) BF image, (**b**,**c**) its corresponding five-fold and two-fold axes SAED patterns, respectively. There also exist some lattice deviations in the five-fold axes SAED pattern because of the liner phason strain. I-phase has a composition of approximate Mg_27.5±3.5_Zn_63.5±1.5_Gd_10±3_ (at.%).

**Figure 7:**
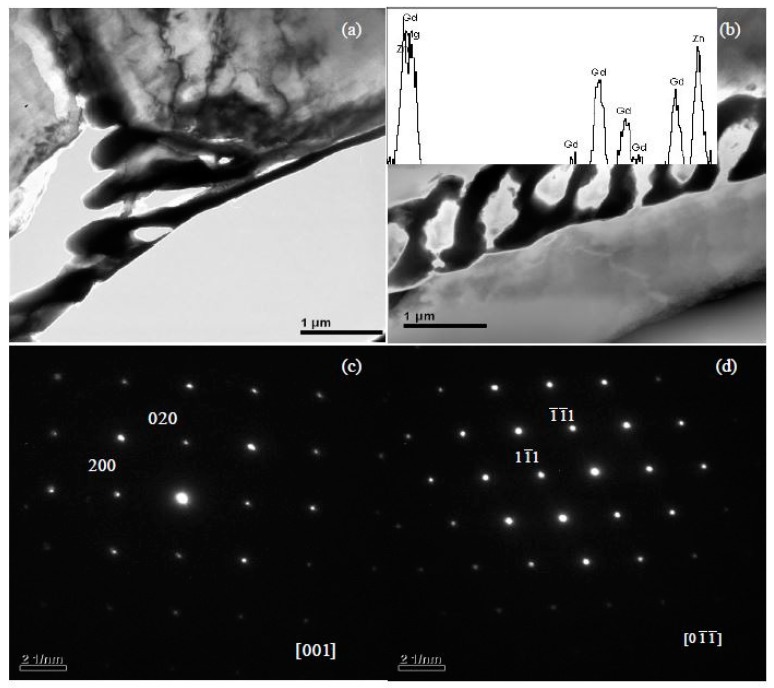
Identification of W-phase with by TEM in Mg-1Zn-1Gd alloy (Sample 36): (**a**) BF image for dendritic shape, (**b**) BF image for twist-like shape, (**c**) SAED pattern along [001] zone axis, and (**d**) SAED pattern along $\left[0\bar{1}\bar{1}\right]$ zone axis. The W-phase in (**b**) has a composition of approximate Mg_36.4_Zn_35.76_Gd_27.84_ (Zn/Gd = 1.3, at.%) and a lattice parameter *a* = 0.7256 nm.

Phases in special alloys were further characterized by TEM. For Mg-5Zn-0.278Gd alloy (Sample 10, Zn/Gd = 18, represented for region (III)), the dendritic phase is identified as I-phase (Figure 4a). A slight lattice deviation from the ideal icosaheral symmetric positions is pointed out with the white arrow in the corresponding selected area electron diffraction (SAED) patterns (Figure 4b), owing to linear phason strains coming from its anisotropic growth during solidification [36]. Through the analysis of EDS, a composition of Mg_30±1_Zn_62_Gd_8±1_ (at.%) is adopted for I-phase. One another phase with triangular shape has also been observed (Figure 4c) and identified as W′-phase by SAED patterns (Figure 4d). It has a composition of approximate Mg_94±1_Zn_5.47±0.5_Gd_0.53±0.1_ (at.%) and a face centered cubic (fcc) structure with a lattice parameter of *a* = 2.3529 nm. The lattice parameter of the W′-phase is triple as one of W-phase [13]. There is a cube-to-cube orientation relationship existing between W-phase and W′-phase, as (${\left(100\right)}_{W}$//${\left(100\right)}_{W^{\prime }}$, ${\left(010\right)}_{W}$//${\left(010\right)}_{W^{\prime }}$, ${\left(001\right)}_{W}$//${\left(001\right)}_{W^{\prime }}$), which has been reported in our previous work [13] and by Luo [37]. W′-phase is usually considered as 2/1 crystalline approximate to I-phase. For Mg-1Zn-0.333Gd alloy (Sample 39, Zn/Gd = 3, for region (II)), the skeleton morphology phase is W-phase (Figure 5a). The corresponding SAED patterns along [001], [111] and [112] zone axes are shown in Figure 5b–d, respectively. The W-phase has a composition of approximate Mg_31_Zn_45_Gd_24_ (Zn/Gd = 1.9, at.%) and it is identified to have a face centered cubic (fcc) structure with a lattice parameter of *a* = 0.6927 nm. The lattice parameter of W-phase in Mg-1Zn-0.333Gd alloy (*a* = 0.6927 nm) is slightly larger than that in Mg-Zn-Y system (*a* = 0.6848 nm) [37]. Meanwhile, I-phase with particle shape is founded (Figure 6). By the extrapolation technique described by Lorimer et al. [38], a composition of Mg_27.5__±3.5_Zn_63.5__±1.5_Gd_10__±3_ (at.%) is adopted to I-phase. For the Mg-1Zn-1Gd alloy (Sample 36, Zn/Gd = 1, represented for region (I)), two kinds of W-phase with dendritic or twist-like shape have been identified in Figure 7a,b. Its corresponding SAED patterns along [001] and $\left[0\bar{1}\bar{1}\right]$ zone axes are shown in Figure 7c,d, respectively. The W-phase in Figure 7b has a composition of Mg_36.4_Zn_35.76_Gd_27.84_ (Zn/Gd = 1.3, at.%) with a lattice parameter of *a* = 0.7256 nm. For W-phase with the cubic structure (Figure 5 and Figure 7), a lower Zn/Gd ratio would result in a larger lattice parameter. W-phase has the similar face-centered cubic structure to Mg_3_Gd ($m\bar{3}m$, *a* = 0.700 nm), and once been considered as Mg_3_Gd [39].

#### 3.1.2. The Effect of Zn/Gd Ratio on the Phase Formation

The effect of Zn/Gd ratio on the phase formation could directly obtained from the Figure 2 and Table 1. The phase formation regions in Mg-rich section are mainly decided by Zn/Gd ratio. When Zn/Gd = 0.25~1.5, the alloys mainly consist of α-Mg and W-phase In the Zn/Gd ratio range of 1.5~6, I-phase is detected as another main phase besides the α-Mg and W-phase. The quasi lattice parameter of the I-phase could be determined as approximately 0.520 nm by the Elser’s method [39], with I-phase indexing from [40]. When Zn/Gd = 6~40, only α-Mg and I-phase exist. I-phase could not be founded in alloys with the Zn/Gd ratio up to 60. Moreover, Mg-5Zn binary alloy (Sample 15) is analyzed for comparison, it contains Mg_2_Zn_3_ and Mg_7_Zn_3_.

**Figure 8:**
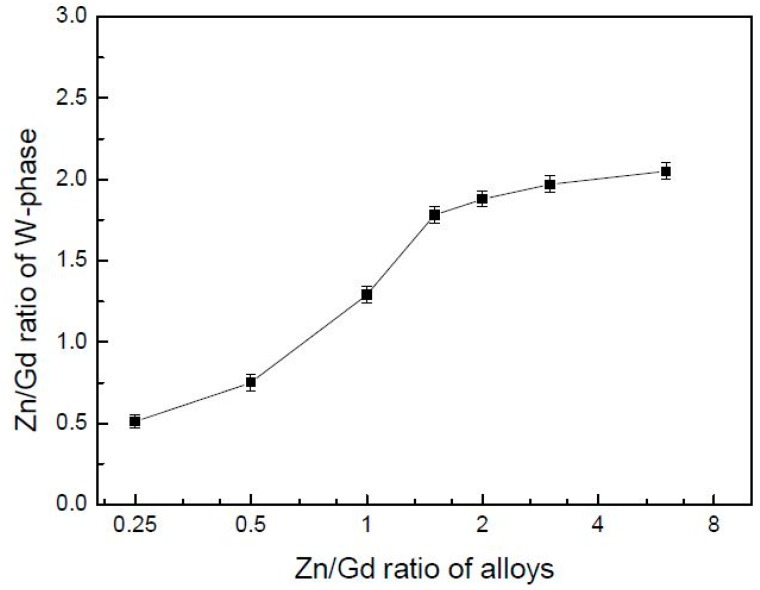
The Zn/Gd ratio in W-phase plotted as a function of Zn/Gd ratio in alloys (Zn/Gd ratio in alloys range from 0.25 to 6, sample 45, 35, 28, 21, 18, 5, 6).

**Figure 9:**
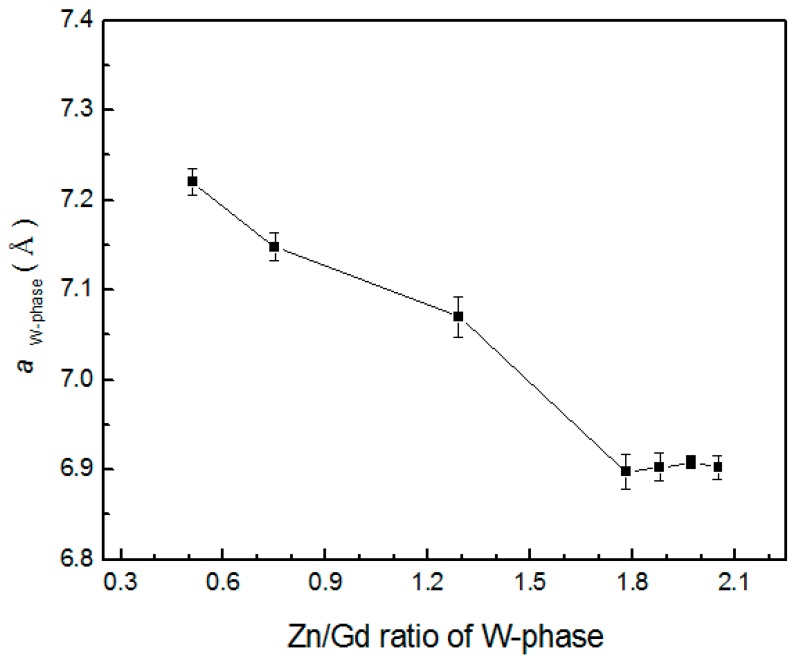
The lattice parameter a W-phase plotted as a function of Zn/Gd ratio in W-phase (Zn/Gd ratio in alloys range from 0.25 to 6, sample 45, 35, 28, 21, 18, 5, 6).

When Zn/Gd ratio of the alloys is between 0.25~6, the W-phase would form, but be of a different composition and with a changed lattice parameter. The Zn/Gd ratio in W-phase is ascribed with the one in alloy (Figure 8), which indicates that extra solute has occurred by the substitute or interstitial mode when W-phase precipitating. The cubic lattice parameter of W-phase could be deduced with the Bragg’s law for the cubic crystal lattice [41]:

(1) $$a=\frac{\lambda }{2\sin\theta }\sqrt{h^{2}+k^{2}+l^{2}}$$

where 2*θ* represents the degree of X-ray peak, *λ* represents the wavelength of Cu-*Kα* radiation, *a* represents the lattice parameter, and (hkl) represent crystal plane for 2*θ*. The mean *a* values of W-phase in alloys have been calculated by six individual X-ray diffraction peaks (corresponding to (111), (200), (220), (311), (400), and (422), Table 3). The lattice parameter *a_W-phase_* deviates with the Zn/Gd ratio in W-phase is shown in Figure 9. It can be concluded that *a_W-phase_* could be a good index for the Zn/Gd ratio of alloys, with more sensitive in low Zn/Gd ratio range (from 0.25 to 1.5) than in the higher Zn/Gd ratio range (from 2 to 6).

**Figure 10:**
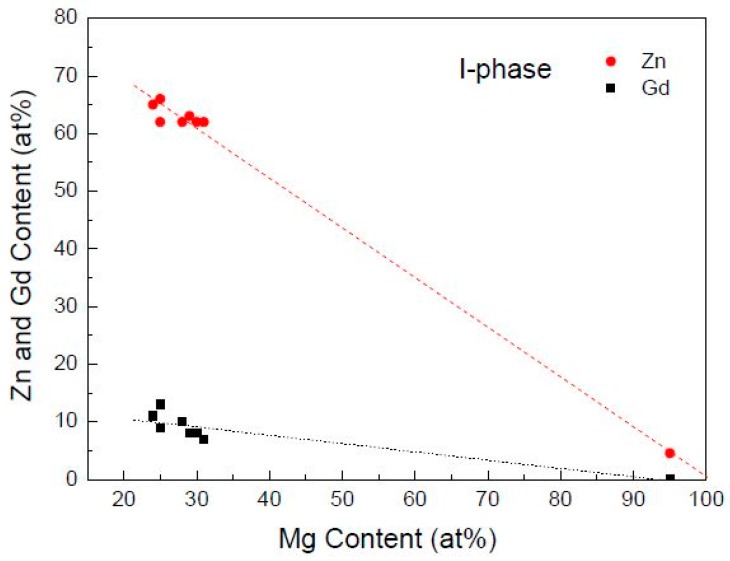
Contents of Zn and Gd of I-phases (Zn/Gd = 6:1) in Mg-1Zn-0.333Gd alloy (Sample 39) plotted as a function of the Mg content by EDS with TEM.

I-phase forms when Zn/Gd ratio in alloys is between 1.5~40, but Zn/Gd ratio in I-phase composition can be faintly effected by it. For example, eight points of I-phase in Mg-1Zn-0.33Gd alloy (Sample 39, Zn/Gd = 3) have confirmed by EDS analysis along with TEM (Figure 10). Although Mg content ranges from 24 to 31, the Zn/Gd ratio is still close to 6, suggesting a standard stoichiometry of Mg_30_(Zn_6_Gd_1_)_70_. Moreover, the composition of I-phase in other alloys, such as Mg-5Zn-0.278Gd (Sample 10, Zn/Gd = 18) or Mg-3Zn-0.3Gd (Sample 23, Zn/Gd = 10), is also quite approximate to the standard stoichiometry. In the other way, some alloys have the same Zn/Gd ratio (Table 4), but with different Zn/Gd ratio in I-phase.

#### 3.1.3. The Effect of Alloying Element Content on the Phase Formation

**Figure 11:**
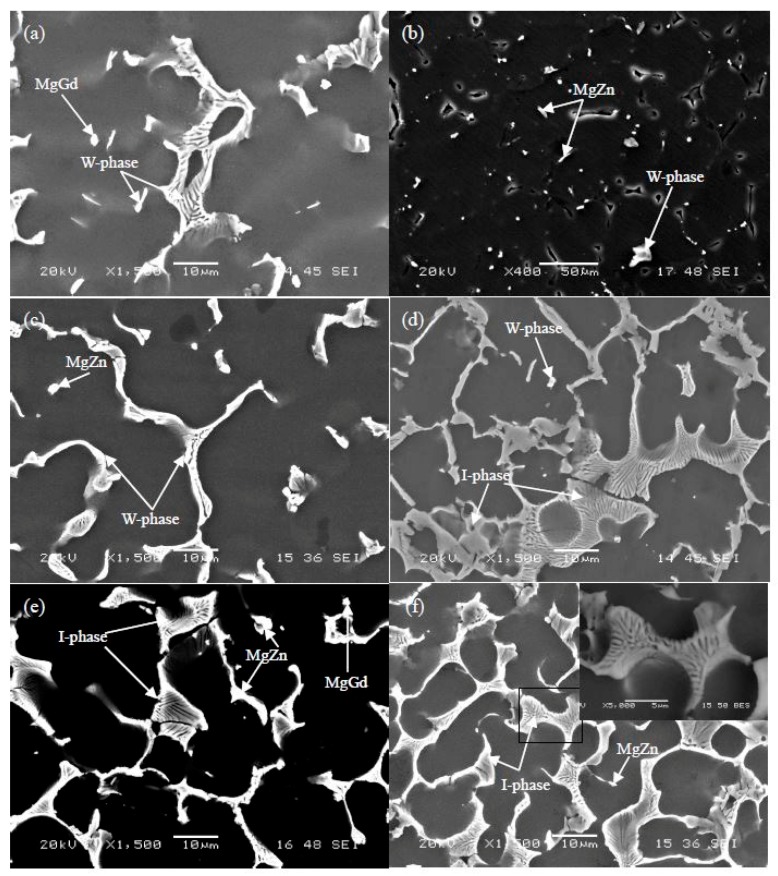
SEI of Mg-Zn-Gd alloys (Zn/Gd ratio, sample No.): (**a**) Mg-0.5Zn-2Gd (0.25, sample 45), (**b**) Mg-1Zn-0.1Gd (10, sample 40), (**c**) Mg-3Zn-0.3Gd (10, sample 23), (**d**) Mg-5Zn-0.5Gd (10, sample 7), (**e**) Mg-10Zn-1Gd (10, sample 1), (**f**) Mg-10Zn-0.4Gd (25, sample 2), and the inset of (**f**) is BSE of rectangle area.

The influence of Zn/Gd ratio on phase formation has been demonstrated above. Furthermore, the alloying element content in alloys plays indispensable roles, as shown in Figure 11. The higher content of alloying elements, under the same Zn/Gd ratio (Figure 11b–e) Zn/Gd = 10), would incline to form more I-phase, together with more binary compounds. This can be mainly associated with the tendency of segregation of residual liquid during solidification.

Mg-Zn-Gd alloy contain is less than 5%. The Gd content effect on microstructure could be gotten from Figure 3 (Sample 35, 29, 10, 24) and Figure 11 (Sample 45, 40, 23, 7, 1, 2). With a higher Gd content, a larger amount of dendritic phase and more closed network microstructure would exist in alloys. While, with a lower Gd content, a dispersed and granular microstructure would exhibit. Gd element is indispensable for I-phase formation. Although the alloy of different composition (Sample 23, 7, 1 with Zn/Gd = 10, and Sample 24, 11, 2 with Zn/Gd = 25), Gd content in these I-phase is approximate to 3 at.% (Table 4).

Since the high Zn content would result in fluidity decreasing, hot tear, and shrinkage porosity, commercial Mg-Zn-Gd alloy contain is less than 10 at.%. The SEI images of Mg-Zn-Gd alloys, in Zn content ascending, are shown in Figure 11. EDS was also carried out. For Mg-0.5Zn-2Gd alloy (Sample 45, Figure 11a), dendritic and strip shaped W-phases (Mg_87.4_Zn_8_Gd_4.6_, at.%, Zn/Gd = 1.74) are found, while the granular particles are confirmed to be Mg_3_Gd. For Mg-1Zn-0.1Gd alloy (Sample 40, Figure 11b), the MgZn particle and gray strip W-phase, in a small amount (not detected by XRD sensitivity), exist besides the I-phase. For Mg-3Zn-0.3Gd alloy (Sample 23, Figure 11c), the dendritic phase is confirmed as I-phase (Mg_75_Zn_21.5_Gd_3.5_, Zn/Gd = 6.14, at.%), while the particle phase in a small amount is verified as MgZn compound (not detected by XRD sensitivity). For Mg-5Zn-0.5Gd alloy (Sample 7, Figure 11d), I-phase exhibits dendritic and lamellar shape morphology having a composition of Mg_67.8–74.7_Zn_22.6–28.6_Gd_2.7–3.6_ (at.%, Zn/Gd = 7.95~8.5). In addition, there exist W-phase particles having a composition of Mg_79.5_Zn_9.6_Gd_10.9_ (at.%). For Mg-10Zn-1Gd alloy (Sample 1, Figure 11e), it contains MgZn and MgGd compounds other than I-phase. For the Mg-10Zn-0.4Gd alloy (Sample 2, Figure 11f), I-phase exhibits block or lamellar shape, having a composition of Mg_55.1_Zn_42.1_Gd_2.8_ (Zn/Gd = 15, at.%). The BSE image of I-phase inset in Figure 11f shows the same contrast, which gives direct evidence for the homogeneity in chemical stoichiometry. The strip MgZn compound appears, as pointed with arrow in Figure 11f.

### 3.2. The DTA of Mg-Zn-Gd Alloys on the Mg-Rich Corner

The DTA analysis was carried out for Mg-Zn-Gd alloys. The DTA analysis of Mg-5Zn alloy (Sample 15) is also given as a comparison benchmark. The liquidus temperature for Mg-5Zn alloy was 613 °C, which agrees with that (610 °C) in the Mg-Zn binary phase diagram [39]. It indicates that there exists little loss of Mg and Zn elements in the melting process, which would just lead to a minor affection to the equilibrium temperature of alloys designed in this work.

**Figure 12:**
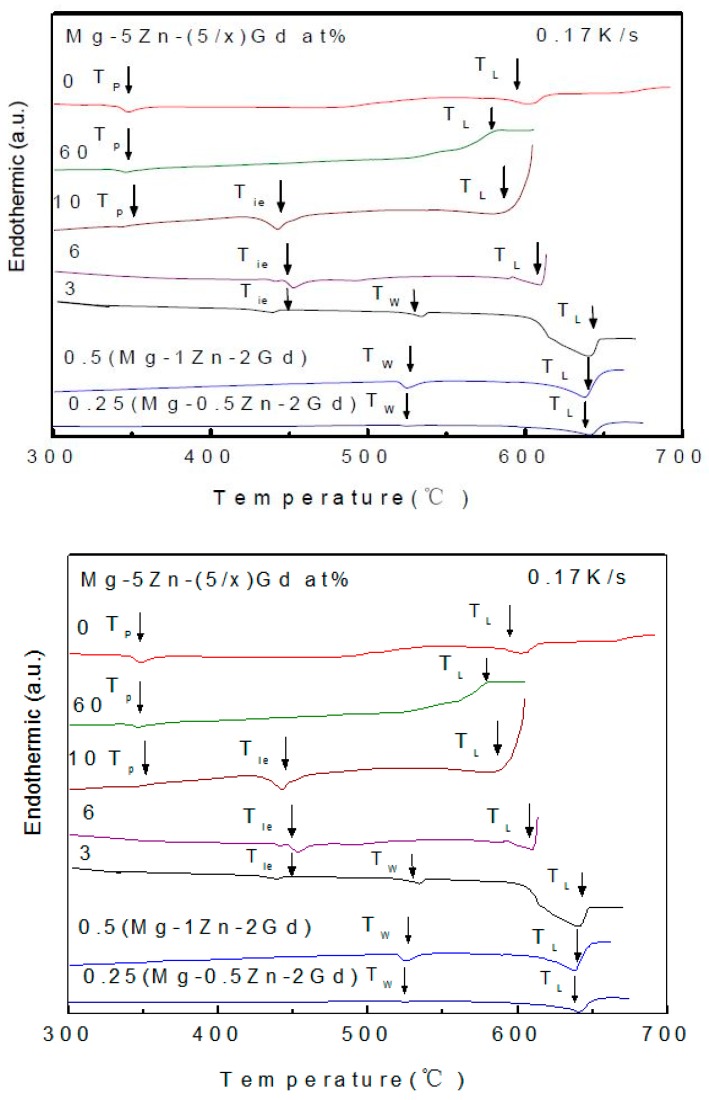
DTA heat trace of Mg-Zn-Gd alloys with 5 at.% Zn (Zn/Gd ratio range from 0.25 to 60, Sample 45, 35, 5, 6, 7, 14, 15).

The characterized temperatures were summarized in Table 5. *T_P_*, *T_ie_*, *T_W_*, and *T_L_*, represent the reaction of binary compound, I-phase, W-phase, and liquidus temperature of alloys, respectively. It should be noted that *T_ie_* and *T_W_* do not vary with the alloy composition change. However, *T_L_* would be quite different. The temperature interval between *T_L_* and *T_ie_* expressed as *ΔT_Li_ = T_L_ − T_ie_*, defined as equilibrium melting range. *ΔT_Li_* would largely affect the I-phase formability. The *T_L_* changing along with the alloy composition would be investigated in details, as follow:

Figure 12 shows the heat trace of Mg-Zn-Gd alloys with different Zn/Gd ratio (Sample 45, 35, 5, 6, 7, 14, 15). As Zn/Gd ≤ 0.5, the Mg-Zn-Gd alloys exhibit similar melting behavior. Two major endothermic peaks are detected at 519 °C (*T_w_*) and 655 °C (*T_L_*). As Zn/Gd = 3, a new endothermic peak at 431 °C (*T_ie_*) emerges other than those for *T_L_* (635 °C) and *T_w_* (519 °C). As Zn/Gd = 6, only endothermic peaks that were associated with *T_L_* (611 °C) and *T_is_* (431 °C) exist. When Zn/Gd = 10, besides endothermic peaks for *T_ie_* (431 °C) and *T_L_* (595 °C), one another endothermic peak at 341 °C (*T_p_*) associated with binary compound emerges. When Zn/Gd = 60, there are only *T_p_* (366 °C) and *T_L_* (588 °C). It could be seen in Figure 12 that *T_L_* decreases as Zn/Gd ratio increasing.

**Figure 13:**
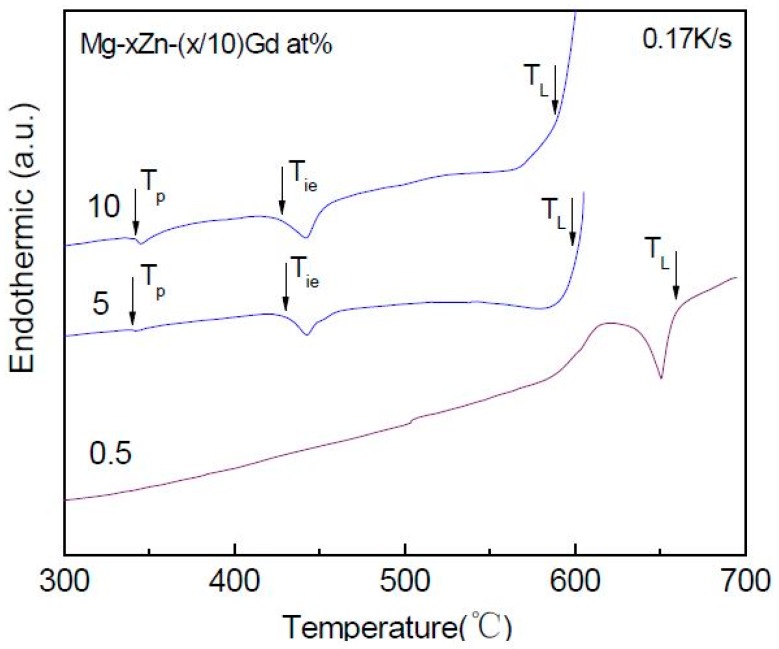
DTA heat trace of Mg-Zn-Gd alloys with Zn/Gd ratio of 10 (Sample 1, 7, 48).

Figure 13 shows the heat trace of alloys with different alloying element content with Zn/Gd = 10 (Sample 1, 7, 48,). Mg-0.5Zn-0.05Gd alloy (Sample 48) only shows one endothermic peak at 655 °C corresponding to *T_L_*. For Mg-5Zn-0.5Gd (Sample 7) alloy and Mg-10Zn-1Gd (Sample 1) alloy two more endothermic peaks are identified, corresponding to *T_p_* (Mg_7_Zn_3_) and *T_is_*, respectively. The endothermic peaks for Mg-10Zn-1Gd alloy are wider than the ones for the Mg-5Zn-0.5Gd alloy. *T_L_* (595 °C) of the former one is higher than *T_L_* (567 °C) of the later one. It could be seen in Figure 13 that *T_L_* decreases as the alloying element content increasing. The same phenomenon could also been found in the alloys with Zn/Gd = 5 in Table 5.

## 4. Discussion

### 4.1. The Structure and Composition of I-Phase

The formation range of I-phase in Mg-Zn-Y system has been reported about in the Zn/Y ratio of 2.7~9.5 (at.%) [42]. While for Mg-Zn-Gd system in present study, the I-phase can be formed in the Zn/Gd ratio range of 1.5~40. It indicates that the Mg-Zn-Gd system has higher formability of I-phase as compared with Mg-Zn-Y system under the conventional cast condition. Furthermore, higher volume fraction of I-phase was formed in alloys that were containing higher alloying element content under same Zn/Gd ratio. As reported previously, the volume fraction of I-phase plays an important role in the properties of alloys, such as Mg-Zn-Y [43] and Mg-Zn-Ho [44]. Singh et al. [19] has dominated strengthening mechanism when I-phase content varied. Meanwhile, our previous results also indicated that the mechanical properties of Mg-Zn-Gd alloys varied with its phase constitutions, such as the I-phase and Laves phase [44]. Therefore, the anticipated mechanical properties of Mg-Zn-Gd alloy can be obtained by adjusted the fraction of I-phase and phase constitution with the help of the diagram, as demonstrated in Figure 2.

Tsai et al. [45] has investigated I-phase in Mg-Zn-RE (RE=Gd, Tb, Dy, Ho, Er) systems using XRD and TEM. The structure type of I-phase in Mg-Zn-RE systems has been classified in the view of quasi lattice parameter and valence electron concentration (e/a). The as-cast alloy with the nominal composition of Mg_42_Zn_5__0_Gd_8_ (at.%) has been proved to be a single I-phase by Tsai et al., but the real stoichiometry of I-phase has still not been detected. The I-phase in Mg-Zn-Gd alloys exhibits a stoichiometry of Mg_30±1_Zn_62_Gd_8±1_ (at.%) in present work, and the average composition (at least three points) of I-phase in each sample was determined by EDS along with TEM or SEM. The icosahedral quasi crystals are generally classified into two groups, depending on the cluster structures [46]. Mackay-icosahedral clusters (MI-type) has a lattice parament of *a* = 4.6 Å and the e/a about 1.75. The rhombic-triacontahedron clusters (FK-type) is the other one, having a quasi lattice of *a* = 5.2 Å and the e/a about 2.1. Table 6 lists the data about e/a, atomic radii, and element electronegativity of I-phase. Zn and Mg element have the same value of e/a about 2, while the Gd element has the value of e/a about 3. Therefore, the content of Gd element plays a significant role in the value of e/a of I-phase. When the content of Gd of I-phase exhibits a relatively settled range of 8 ± 1 at.%, it would result in the e/a value between 2.06 to 2.1, which was nearly matching with the FK-type. In the view of atomic size, the value of δ (δ = (r_A_ − r_B_)/r_A_) for Zn/Mg is about 16.8%, which exceeded the limits of the solubility. The literature [44] has reported that the 5 at.% Zn replacement by Mg in Mg-Zn-Y quasicrystalline phase would induce an expansion of the lattice, which confirmed the dissolution of Zn in the quasicrystalline phase. This means that the replacement between Mg and Zn in I-phase has been restricted. In the aspect of electronegativity, Zn, Mg, and Gd is about 1.65, 1.31, and 1.1, respectively. The large difference in electronegativity among Mg, Zn, and Gd, gives an indirect evidence of the high stability of I-phase. Since the configuration of I-phase can be elucidated with valence electron concentration, atomic size, and electronegativity, the formation and stability of I-phase could be understood within the frame work of the Hume-Rothery rules. However, it is still unclear that why the chemical stoichiometry of I-phase would be different, i.e., formed during casting processing (Mg_30±1_Zn_62_Gd_8±1_, at.%), nano-size formed during hot extrusion (Mg_40_Zn_52_Gd_8,_ at.%) [13]. The equilibrium solidification range *ΔT_Li_* changes with the composition of alloying (Table 5), and *ΔT_Li_* would largely affect the I-phase formability. J. Grobner, et al. [34] also pointed out that the formation of I-phase should largely depend on the composition range of alloys with certain thermodynamic conditions. However the nucleate kinetics of I-phase still needs to be studied further.

### 4.2. The Structure and Composition of W-Phase

**Figure 14:**
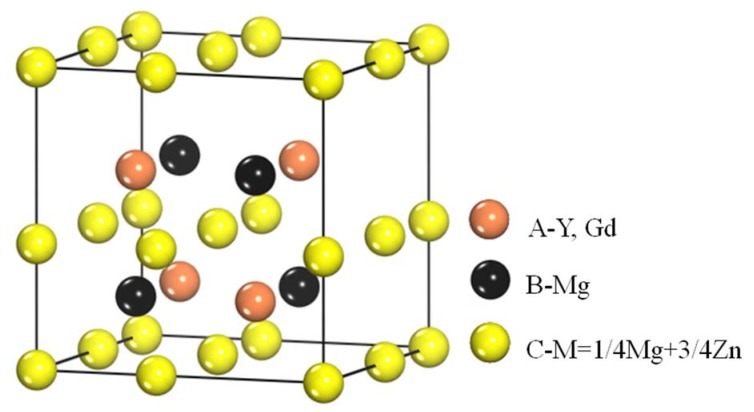
The crystal structure of the W phase in the Mg-Zn-Y(Gd) system, the atomic coordinates.

The W-phase with *fcc* structure in Mg-Zn-Gd system can be formed in the Zn/Gd ratio range of 0.25–6. This kind of W-phase was firstly determined by E. M. Padezhnova et al. [28] by X-ray diffraction in the Mg-Zn-Y system. It possesses a partially ordered AlMgCu_2_-type *fcc* structure: *a* = 0.6848 nm and its space group is $m\bar{3}m$ [37]. The crystal structure of the W-phase in the Mg-Zn-Y system was shown in Figure 14. It can be found that four Y atoms occupy the 4A positions, four Mg atoms occupy the 4B positions, and eight (Mg+Zn) atoms, with the ratio of 1:3, occupy the 8C positions. When Gd atom instead of Y atom, the W-phase exhibits a larger lattice value due to the larger atomic size (r_Gd_ = 0.181 nm, r_Y_ = 0.178 nm). Meanwhile, the lattice parameter *a* of the W-phase is very sensitive to the composition of Mg-Zn-Gd alloys, especially when affected by the Zn/Gd ratio of alloys. The Gd solution would induce the lattice expansion. W-phase would transform into another compound under certain condition by the diffusion and rearrangement. This has been verified by its disappearance and transformation to the 14H phase during the heat-treatment at 773 K, as reported by M. Yamasaki [26]. The similar phenomenon occurred in the Mg-Zn-Y system [23,28]. The X-phase with 18R-type structure, in as-cast Mg-Zn-RE-Zr alloy with low content of Zn and high content of RE, transformed into the 14H LPSO structure through certain heat treatment procedure [47]. In addition, the occurrence of W-phase in Mg-Zn-Gd alloy indirectly reveals that W-phase would transform under some condition.

The study of Mg-Zn-Y alloys [48,49,50,51] indicated that the existence of W-phase was unfavorable to the mechanical properties of as-extruded alloys. Thus, the formation range of the W-phase should be avoided during the design of I-phase strengthening Mg-Zn-Gd as-extruded alloys.

## 5. Conclusions

The formation range of I-phase and W-phase of Mg-Zn-Gd alloy in the Mg-rich section has been explored. The effect of Zn/Gd ratio and the alloying element content on phase constituents has been systematically elucidated. Results that were obtained in the present work can be summarized, as follows:

(1) Five regions can be classified in this Mg-rich section: (I)α-Mg+W-phase(+binary compounds), (II)α-Mg+W-phase+I-phase(+binary compounds), (III)α-Mg+I-phase (+binary compounds), (IV)α-Mg+binary compounds, and (V)α-Mg. This diagram of phase constitution would give a guideline for the design of Mg-Zn-Gd alloys to match the expecting properties by obtaining the desired phase components.
(2) The I-phase in Mg-Zn-Gd alloys has a composition of Mg_30±1_Zn_62_Gd_8±1_ (at.%), belonging to FK-type quasicrystalline phase in terms of the Hume-Rothery rules. I-phase can be formed in Zn/Gd ratio range of 1.5~40. The equilibrium solidification range of *ΔT_Li_* is boarded when Zn/Gd ratio or alloying element content decrease, and it would largely influence the formability of the I-phase.
(3) The W-phase in Mg-Zn-Gd alloy has *fcc* structure with the space group $m\bar{3}m$. It can be formed only in the Zn/Gd ratio range of 0.25~6. The composition of the W-phase is very sensitive to the composition of Mg-Zn-Gd alloys.

## Figures and Tables

**Table 1:** The summary of the phase constitutions in Mg-Zn-Gd alloy with different compositions.

No.	Composition (at.%)	Zn/Gd Ratio	Experimentally Detected Phases (Other than α-Mg) by XRD	Analysis Beside XRD	Regions	Ref.
	Mg-19Zn-1Gd	19.00	I+MgZn		III	[34]
	Mg-17.5Zn-2.5Gd	7.00	I+W		II	[34]
	Mg-16Zn-4Gd	4.00	I+W		II	[34]
	Mg-14Zn-6Gd	2.33	I+W		II	[34]
	Mg-14Zn-4.9Gd	2.86	I+W		II	[34]
	Mg-11Zn-9Gd	1.22	W		I	[34]
1	Mg-10Zn-1Gd	10.00	I+W+MgZn_2_	DTA/SEM	II	
2	Mg-10Zn-0.4Gd	25.00	I+Mg_2_Zn_3_	EDS/SEM	III	
3	Mg-8Zn-0.8Gd	10.00	I+W+Mg_2_Zn_3_		II	
4	Mg-8Zn-0.32Gd	25.00	I+MgZn_2_		III	
	Mg-5Zn-15Gd	0.33	Mg_3_Gd		IV	[34]
5	Mg-5Zn-1.667Gd	3.00	I+W	DTA	II	
6	Mg-5Zn-0.833Gd	6.00	I+W	DTA	II	
7	Mg-5Zn-0.5Gd	10.00	I	EDS/DTA/SEM	III	
8	Mg-5Zn-0.417Gd	11.99	I		III	
9	Mg-5Zn-0.333Gd	15.02	I		III	
10	Mg-5Zn-0.278Gd	17.99	I	EDS/SEM /TEM/SAED	III	
11	Mg-5Zn-0.2Gd	25.00	I	EDS	III	
12	Mg-5Zn-0.125Gd	40.00	I		III	
13	Mg-5Zn-0.125Gd	40.00	I+Mg_2_Zn_5_		III	
14	Mg-5Zn-0.083Gd	60.24	Mg_2_Zn_3_+Mg_7_Zn_3_	EDS/DTA/SEM	IV	
15	Mg-5Zn		Mg_2_Zn_3_+Mg_7_Zn_3_	DTA	IV	
16	Mg-4.8Zn-0.4Gd	12.00	I		III	
17	Mg-4.2Zn-0.8Gd	5.25	I		III	
18	Mg-4Zn-2Gd	2.00	I+W		II	
19	Mg-4Zn-1.333Gd	3.00	I+W		II	
20	Mg-3.5Zn-0.6Gd	5.83	I		III	
21	Mg-3Zn-2Gd	1.50	I+W		II	
22	Mg-3Zn-1.5Gd	2.00	I+W		II	
23	Mg-3Zn-0.3Gd	10.00	I	EDS/SEM	III	
24	Mg-3Zn-0.12Gd	25.00	I	EDS	III	
25	Mg-2.8Zn-0.5Gd	5.60	I		III	
26	Mg-2.5Zn-2Gd	1.25	W		I	
27	Mg-2.25Zn-0.75Gd	3.00	I+W		II	
28	Mg-2Zn-2Gd	1.00	W		I	
29	Mg-2Zn-1.5Gd	1.33	W		I	
30	Mg-2Zn-1Gd	2.00	W		I	
	Mg-2Zn-1Gd	2.00	14H+Mg_5_Gd		IV	[27]
31	Mg-2Zn-0.1Gd	20.00	I		III	
	Mg-1.55Zn-0.937Gd	1.65	W+X(LPSO)+Mg_3_Gd		I	[32]
	Mg-1.51Zn-2.78Gd	0.54	W+Mg_3_Gd		I	[32]
32	Mg-1.5Zn-1Gd	1.50	W		I	
33	Mg-1.2Zn-0.2Gd	6.00	I+W		II	
34	Mg-1.2Zn-0.1Gd	12.00	I		III	
35	Mg-1Zn-2Gd	0.50	W	EDS/DTA/SEM	I	
36	Mg-1Zn-1Gd	1.00	W	TEM/SAED	I	
37	Mg-1Zn-0.667Gd	1.50	W		I	
38	Mg-1Zn-0.5Gd	2.00	W		I	
	Mg-1Zn-0.5Gd	2.00	14H+Mg_3_Gd		IV	[33]
39	Mg-1Zn-0.333Gd	3.00	I+W+Mg_5_Gd	EDS/SEM /TEM/SAED	II	
40	Mg-1Zn-0.1Gd	10.00		SEM	V	
41	Mg-1Zn-0.04Gd	25.00			V	
42	Mg-0.75Zn-1Gd	0.75	W		I	
43	Mg-0.6Zn-0.2Gd	3.00			V	
44	Mg-0.6Zn-0.2Gd	3.00	I+W		II	
45	Mg-0.5Zn-2Gd	0.25	W+Mg_3_Gd	DTA/SEM	I	
46	Mg-0.5Zn-1Gd	0.50	W		I	
47	Mg-0.5Zn-0.5Gd	1.00	W		I	
48	Mg-0.5Zn-0.05Gd	10.00		DTA	V	
	Mg-0.283Zn-0.796Gd	0.36	W+Mg_3_Gd		I	[32]
	Mg-0.276Zn-2.48Gd	0.11	I+W+Mg_3_Gd		II	[32]

**Table 2:** Energy-dispersal X-ray spectroscopy (EDS) analysis of the compounds in Mg-Zn-Gd alloys along with scanning electron microscopy (SEM) (D-Dendritic, P-Particle, M-Matrix).

Alloys (Zn/Gd Ratio)	Location	Mg at.%	Zn at.%	Gd at.%	Zn/Gd Ratio	Compound Remarks	
Mg-1Zn-2Gd (0.5)	D	90.87	3.95	5.19	0.76	MgZnGd	Sample 35
	P	93.47	2.78	3.75	0.74	MgZnGd	
	M	99.29	0.20	0.51	0.39	Gd-rich	
Mg-1Zn-0.333Gd (3)	D	79.10	13.88	7.03	1.97	MgZnGd	Sample 39
	P	83.09	0.62	16.3		Mg_5_Gd	
	M	99.34	0.66			Zn-rich	
Mg-5Zn-0.278Gd (18)	D	56.02	40.03	3.94	10.2	I-phase	Sample 10
	P	91.93	8.07			Mg_2_Zn_3_	
	M	96.43	3.34	0.23		Zn-rich	
Mg-5Zn-0.083Gd (60)	D	82.88	16.35	0.77	21.2	Mg_2_Zn_3_ with Gd	Sample 14
	P	73.91	18.17	7.92	2.29	MgZnGd	
	M	98.21	1.79			Zn-rich	

**Table 3:** The mean values of a_W-phase_ in Mg-Zn-Gd alloys through 2θ of peaks in X-ray diffraction (XRD) patterns (Zn/Gd ratio in alloys range from 0.25 to 6).

Alloys (Zn/Gd Ratio)	Crystal Plane (*hkl*)						Mean *a* Value	
	(111)	(200)	(220)	(311)	(400)	(422)	(*Å*)	
Mg-0.5Zn-2Gd (0.25)	7.217	7.230	7.217	7.215	7.221	—	7.220 ± 0.015	Sample 45
Mg-1Zn-2Gd (0.5)	7.144	7.155	7.146	7.145	7.158	7.142	7.148 ± 0.016	Sample 35
Mg-2Zn-2Gd (1)	7.066	7.088	7.065	7.068	7.065	7.067	7.070 ± 0.023	Sample 28
Mg-3Zn-2Gd (1.5)	6.909	6.904	—	6.892	6.894	6.890	6.898 ± 0.019	Sample 21
Mg-4Zn-2Gd (2)	6.909	6.904	—	6.901	6.894	6.906	6.903 ± 0.015	Sample 18
Mg-5Zn-1.67Gd (3)	6.909	6.909	—	6.907	6.903	6.910	6.908 ± 0.007	Sample 5
Mg-5Zn-0.83Gd (6)	6.909	6.904	—	—	6.897	—	6.903 ± 0.013	Sample 6

**Table 4:** EDS analysis of I-phase in Mg-Zn-Gd alloys by using SEM.

Alloys (Zn/Gd Ratio)	I-Phase (at.%)				
	Mg	Gd	Zn	Zn/Gd Ratio	
Mg-3Zn-0.3Gd (10)	75.28	3.47	21.24	6.12	Sample 23
Mg-5Zn-0.5Gd (10)	67.78	3.61	28.61	7.93	Sample 7
Mg-10Zn-1Gd (10)	65.69	3.71	30.60	8.25	Sample 1
Mg-3Zn-0.12Gd (25)	70.63	2.46	26.92	10.94	Sample 24
Mg-5Zn-0.2Gd (25)	62.87	3.86	33.28	8.62	Sample 11
Mg-10Zn-0.4Gd (25)	55.11	2.79	42.10	15.09	Sample 2

**Table 5:** Characteristic temperatures for Mg-Zn-Gd alloys in the differential thermal analysis (DTA) curves.

Alloys (Zn/Gd Ratio)	Characteristic Temperatures (°C ± 0.2)					
	*T_P_*	*T_ie_*	*T_W_*	*T_L_*	*T_L_-T_i_*	
Mg-0.5Zn-2Gd (0.25)			519	635		Sample 45
Mg-1Zn-2Gd (0.5)			519	635		Sample 35
Mg-5Zn-1.667Gd (3)		431	519	635	204	Sample 5
Mg-5Zn-0.833Gd (6)		431		611	180	Sample 6
Mg-5Zn-0.5Gd (10)	341	431		595	164	Sample 7
Mg-5Zn-0.083Gd (60)	336			588		Sample 14
Mg-5Zn	336			613		Sample 15
Mg-0.5Zn-0.05Gd (10)				655		Sample 48
Mg-10Zn-1Gd (10)	340	428		567	139	Sample 1

**Table 6:** Data of the valence electron concentration (e/a), Atomic radii (a) and Electro negativity of elements involved in I-phase.

Elements	Valence Electron Concentration (e/a)	Atomic Radii (nm)	Electro Negativity	Ref
Mg	2	0.160	1.31	
Zn	2	0.137	1.65	
RE (Gd, Tb, Dy, Ho, Er)	3			[45]
Gd	3	0.181	1.1	[44,45,46]
Y	3	0.178	1.2	[42,43]
